# Retroperitoneal space-occupying lesion with displacement of the inferior vena cava

**DOI:** 10.1016/j.ijscr.2019.03.037

**Published:** 2019-03-30

**Authors:** Stefan Niebisch, Holger Staab, Sebastian Ullrich, Karl-Titus Hoffmann, Osama Sabri, René Thieme, Katrin Schierle, Christian Wittekind, Ines Gockel

**Affiliations:** a Department of Visceral, Transplant, Thoracic and Vascular Surgery, University Hospital of Leipzig, Leipzig, Germany; b Department of Diagnostic and Interventional Radiology, University Hospital of Leipzig, Leipzig, Germany; c Department of Nuclear Medicine, University Hospital of Leipzig, Leipzig, Germany; d Institute of Pathology, University Hospital of Leipzig, Leipzig, Germany

**Keywords:** Retroperitoneal space-occupying soft-tissue tumor, Retroperitoneal schwannoma, Imaging modalities, Preoperative biopsy, Surgical strategy

## Abstract

**Introduction:**

The differential diagnosis and therapy of retroperitoneal masses suspicious for soft tissue tumors remains difficult and needs individual decisions as to the best management of patients. We report an unusual case of retroperitoneal schwannoma (RS).

**Presentation of case:**

We report on a 57-year-old female patient with a retroperitoneal space-occupying lesion with displacement, but no infiltration of surrounding vessels, especially the inferior vena cava. As presence of malignancy could not be completely excluded by imaging and biopsy, we performed an open resection of the tumor with plastic reconstruction of the right renal vein. Curative resection of the tumor could be achieved and benign RS verified.

**Discussion:**

Preoperative work-up, including a broad spectrum of differential diagnoses, and consecutive appropriate surgical resection are challenging. Characteristic features of the pre-therapeutic diagnostics of retroperitoneal schwannomas are discussed and differential diagnostic considerations as well as surgical therapeutic options are outlined.

**Conclusion:**

Deeper understanding of retroperitoneal schwannomas gained from preoperative radiologic diagnostics may serve to underline the need for targeted biopsy. This and its histopathological examination necessitate expert interventional radiologists and pathologists. Thus, even the pre-therapeutic diagnostic management should be performed in a specialized center. If doubts remain regarding the entity of the retroperitoneal lesion and findings are still controversial after thorough assessment, surgical oncologic resection represents the therapy of choice, if the surgical risk is adequate.

## Highlights

• Exact preoperative diagnosis and surgery of retroperitoneal schwannoma (RS) is sophisticated.
• Due to heterogeneity of the entity, preoperative negative biopsy does not exclude malignancy.
• A curative (R0) resection must be achieved in order to avoid local recurrence, always under consideration of multivisceral resections.

## 1 Introduction

Primary retroperitoneal soft-tissue tumors are rare and heterogeneous with view to histology and malignant potential [1]. Soft-tissue tumors are mostly of mesenchymal (more rarely of neuroectodermal) origin and account for approx. 1% of all malignant diseases. Current data indicate a slight downward tendency over the past few years [2]. While soft-tissue sarcomas arise most commonly (60%) in the lower and upper extremities, retroperitoneal and intraperitoneal (visceral) organs represent the second (20%) most frequent location for the development of this entity [3].

A conclusive pre-therapeutic radiologic differentiation between benign and malignant retroperitoneal space-occupying lesions is challenging and not always possible, even though imaging criteria as, e.g. the configuration of the borderline of a space- occupying lesion with regard to the surrounding tissue, or of their internal structure(s) may give rise to the suspicion of a specific lesion [4]. In accordance with Clinical Guidelines, the pre-therapeutic biopsy remains an important tool in achieving an accurate histologic diagnosis. The extent of surgical resection and the possible initiation of neoadjuvant therapy or, respectively, the exclusion of space-occupying masses unsuitable or contraindicated for resection (e.g. retroperitoneal lymphoma, extragonadal seminoma) can only be assessed based on the results of the pre- therapeutic, image-guided biopsy [5]. Retroperitoneal schwannomas (RS) account for only 1–3% of all schwannomas, and for merely 1% of all retroperitoneal tumors [6]. The pre-therapeutic biopsy has to be highly precise for these tumor entities due to their intralesional heterogeneity with transition to an infiltrative malignant growth pattern, which might not be representative for the whole tumor. Thus, it is of diagnostic value only, if the image-guided biopsy is carried out by a sophisticatedly trained interventional radiologist and histopathological examination is performed by a specialized pathologist. An interdisciplinary discussion at a center with extensive experience in the treatment of sarcomas is therefore essential prior to the biopsy procedure. The case has been reported in line with the SCARE criteria [7].

## 2 Case report

**Fig. 1:**
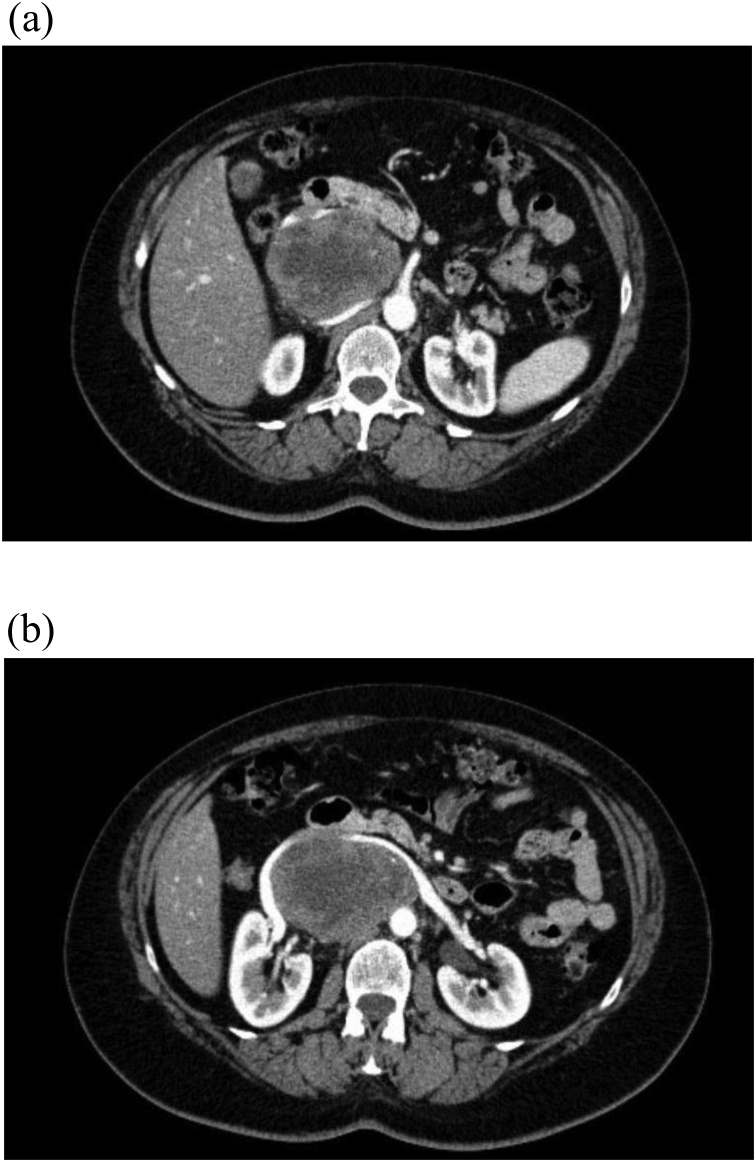
The CT examination (after intravenous contrast administration, transverse section) shows a large, retroperitoneal space-occupying lesion right para-aortal, causing marked ventral displacement of the inferior vena cava, the right renal vein and the adjacent duodenum. Partially visible calcification left ventral within the additionally inhomogeneous lesion as the image morphologic correlate of histopathologically partially identifiable marked regressive changes.

**Fig. 2:**
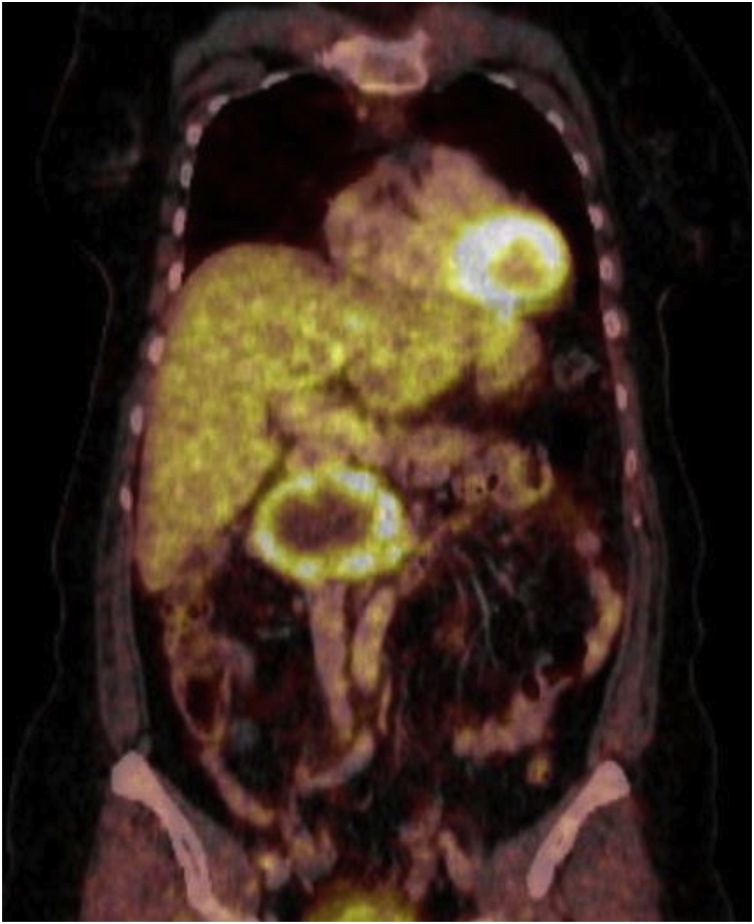
[^18^F]FDG-PET/CT confirmed the presence of a lateral space-occupying lesion para-aortal right at the level of the renal hilus with a malignoma-typical increase in metabolic activity in the marginal areas.

A 57-year-old female patient was referred to our hospital for further evaluation of the accidental finding of a space-occupying lesion in the upper right abdomen made in the course of a sonographic evaluation of the underlying cause of upper abdominal pain. Although the patient did not report unintentional weight loss, she complained of the presence of night sweats unaccompanied by fever over the past 12 months, as well as of the occurrence of exertional dyspnea. There were no significant pre- existing disorders, and neurofibromatosis had not been diagnosed. The CT-scan showed an axial space-occupying lesion of approximately 9.1 x 6.6 cm right-sided paramedian retroperitoneal, with displacement of the inferior vena cava and stretching of the right renal vein (Fig. 1**A**/**B**). [18 F]FDG-PET/CT confirmed the presence of the described right lateral paraaortic lesion, situated at the level of the renal hilus with a malignoma-typical increase of metabolic activity in the marginal areas (Fig. 2). No hematogenic or lymphogenic metastases were identified. Pheochromocytoma was excluded by negative catecholamines and metanephrines in the 24h-urine collector. The punch cylinders obtained on CT-guided puncture were in some instances characterized by hyalinized and cell-poor spindle cell proliferation, while siderite pigmentation was observed others. Immunohistochemically, the spindle cells were strongly positive for S-100 and showed nuclear positivity for SOX-10 at negativity for MDM2, Desmin, CD1a, Melan-A, and HMB-45, as well as for smooth muscle actin (SMA). The MIB-1 proliferation index ranged at < 1%. Molecular-biologic analysis did not yield evidence of MDM2 amplification. The overall histopathologic assessment of the described factors leads to the diagnosis of a benign schwannoma with marked regressive changes.

**Fig. 3:**
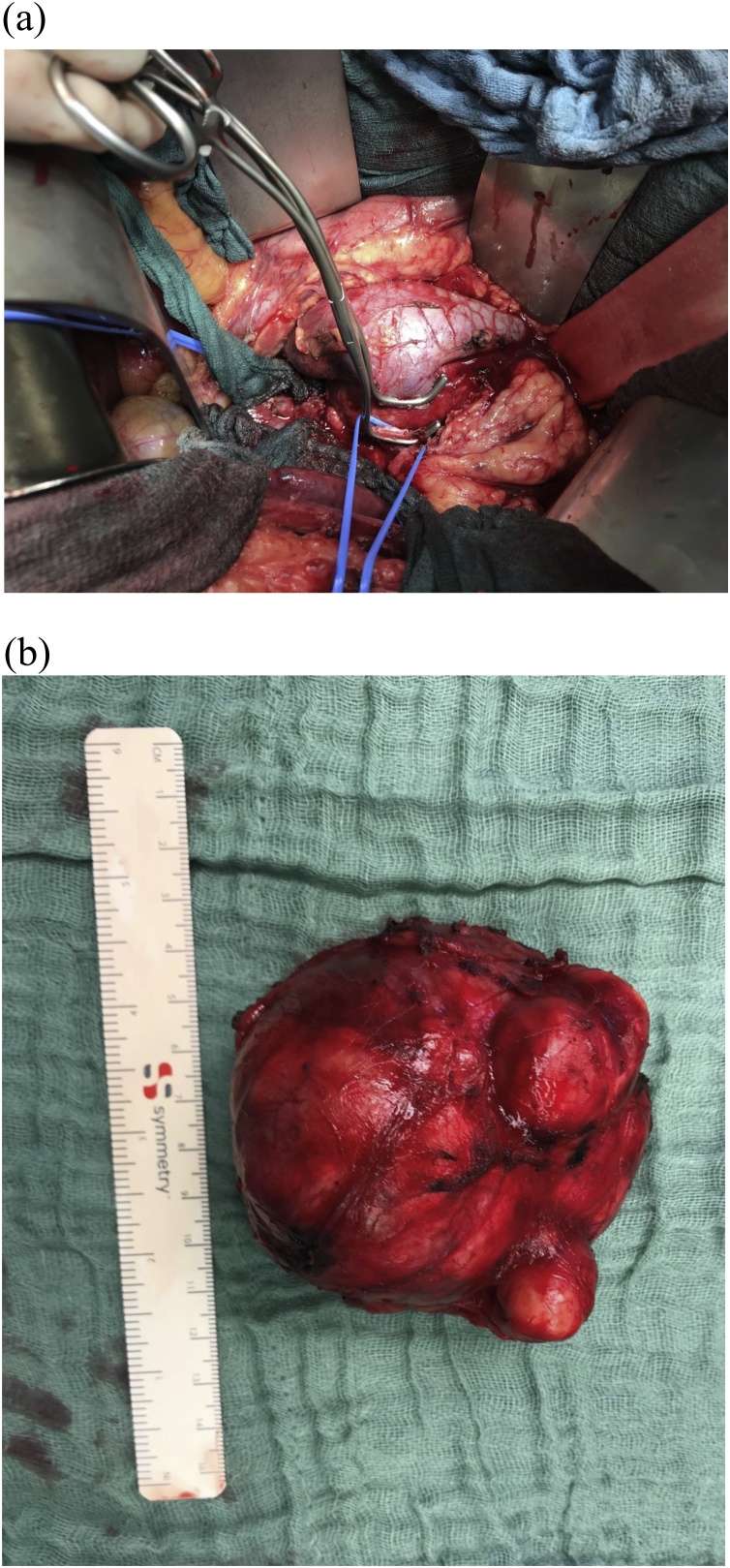
**A**) Intraoperative situs: We performed an open resection of the tumor with plastic reconstruction of the right renal vein (blue vessel loop and Satinski clamp: right renal vein). **B**) Macroscopy of the *en bloc*-specimen revealed a solid, greyish, spherical tumor with a soft capsule and a heterogeneous center. The surrounding “pseudocapsule” was completely removed. Curative (R0) resection was confirmed.

**Fig. 4:**
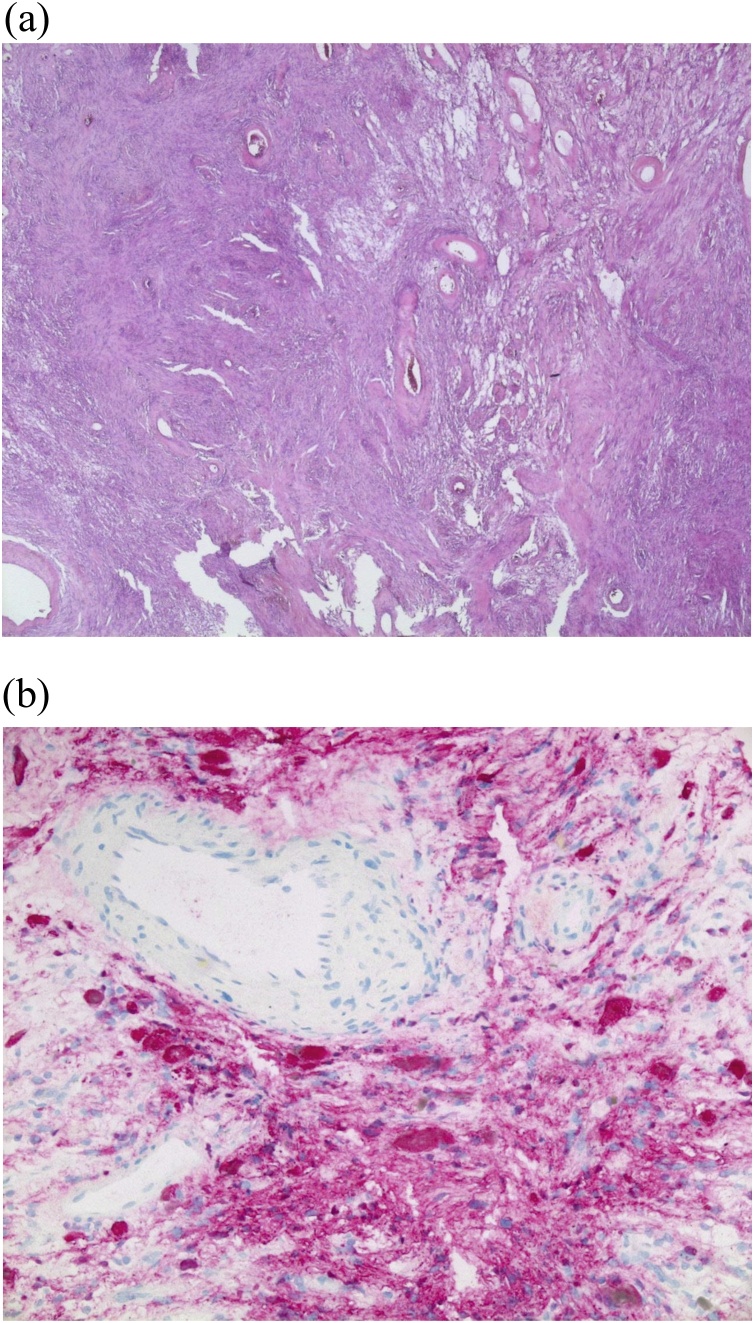
**A**) HE – 25x: Overview image of the resected tissue with richly vascular areas and myxoid-regressive alterations at the image center. **B**) S100 – 200x: Immunohistochemistry for S100 with positive tumor cells and negative vessels obtained by punch-assisted biopsy.

The observed displacement of both, the inferior vena cava and the right renal vein, as well as the malignoma-typical increase of metabolic activity in the [18 F]FDG- PET/CT led to the assumption – in spite of negative histology - that the presence of malignancy could not be excluded. Consecutively, we performed an open resection of the tumor with plastic reconstruction of the right renal vein, while the inferior vena cava could be completely preserved, as it was unaffected (Fig. 3A). Macroscopy revealed a solid, greyish, spherical tumor with a soft capsule and a heterogeneous center (Fig. 3B). The final histopathologic examination confirmed the presence of a partially myxoid schwannoma of 8.5 cm on the largest diameter, showing significant regressive changes and a partially cystic component, as well as evidence of metaplastic ossification (Fig. 4**A**/**B**). The tumor together with the intact capsule was resected curatively (R0-resection). The postoperative course was uncomplicated and the patient was discharged from hospital on postoperative day 8. No adjuvant therapy was indicated and regular aftercare only was recommended by the interdisciplinary tumor board.

## 3 Discussion

Approximately 90% of schwannomas occur solitarily and sporadically, while about 4% of these are associated with neurofibromatosis type 2 [[8], [9], [10]]. The clinical characteristics are nonspecific, dependent upon size, localization, and possible displacement of neighboring organs or structures in the case of benign schwannomas, while malignant ones are subject to alterations due to infiltration of these organs or structures.

Differential diagnosis is challenging – and the meticulous assessment, whether to operate or to pursue conservative management - is to be outlined in our report. Specific laboratory or tumor markers do not exist. The detection of a retroperitoneal space-occupying lesion in the course of an ultrasound examination for clarification of upper abdominal pain, as performed in our patient, is the exception rather than the rule. Frequent CT-morphologic findings include circumscribed round and oval tumors, or spindle-shaped soft-tissue tumors with a lower density than, e.g. muscle tissue, which is characterized by a higher myelin and consequently also by a higher lipid content [11]. Although the relationship between the tumor and the intervertebral foramen is of importance to support the diagnosis of retroperitoneal schwannoma, the absence of this relationship does not necessarily serve to exclude the diagnosis of schwannoma [12]. In our patient, the radiologically visible partial calcifications of the inhomogeneous space-occupying lesion represented the image morphologic correlate of subsequently histopathologically confirmed and partly pronounced regressive changes. Computed tomography may demonstrate further degenerative modulations as, e.g. hemorrhages and hyalinizations [13]. Of particular note in our patient was the displacement of the inferior vena cava, the right renal vein, and the neighboring duodenum in a markedly ventral direction, although imaging did not demonstrate the presence of invasive growth in the sense of direct tumor infiltration. The “target phenomenon” seen on T2-weighted MR imaging as a hyperintense rim surrounding a hypointense central area is a characteristic sign of schwannomas [14]. Radiologic modalities are unfortunately not able to differentiate conclusively between benign and malignant findings, unless clear tumor infiltration or distant metastasis can be clearly detected [8]. The association between an increased [^18^F] FDG uptake by non-gastrointestinal schwannomas and tumor size, cellularity as well as microvascular density in the [^18^F]FDG-PET or [^18^F]FDG-PET/CT has been controversially discussed [15,16]. In the case of our patient, the [^18^F]FDG-PET/CT scan confirmed the presence of a para-aortic lesion located on the right, at the level of the renal hilus with a malignoma-typical increase in metabolic activity in the border region. There was no evidence for metastatic spread.

Both, the image-supported biopsy and the histopathologic evaluation therefore were of utmost relevance for further pre-therapeutic diagnosis. In the performance of a differential diagnosis, consideration should be given not only to space-occupying lesions of neighboring retroperitoneal organs and structures, in particular to those arising from the adrenal glands, such as pheochromocytoma, the pancreas, the sympathetic trunk (e.g. paragangliomas), or the inferior vena cava (e.g. leiomyosarcoma), but potential hemato-oncologic conditions (e.g. lymphoma) or retroperitoneal, extragonadal seminoma also require careful evaluation as differential diagnoses. In all of the described instances and, in particular in the presence of the above mentioned suspected diagnoses, the performance of a pre-therapeutic biopsy is an essential prerequisite, even in cases of apparent resectability, to avoid the (primary) surgical intervention. However, preoperative biopsy is contraindicated, if pheochromocytoma is suspected.

Although findings were controversial in our patient, we decided to operate. The lesion should always be resected oncologically, if there are any doubts of malignancy left and the surgical risk is adequate. In view of the poor response of retroperitoneal schwannomas to chemo- or radiation therapy, primary surgery represents the procedure of choice. The surgical therapy always requires complete removal of the tumor, inclusive of the capsule and the associated pseudocapsule (neovascularization zone). The described removal is also indispensable to prevent the development of local recurrence in cases where the preoperative biopsy does not permit the definitive exclusion of malignity. In our patient, the inferior vena cava was displaced, but not infiltrated and the tumor was dissected without complications. The right renal vein required reconstructive plastic surgery, which eliminated the need for a simultaneous en-bloc resection of the respective vessels and the right kidney itself. Postoperative aftercare is indicated, in particular since the incidence of metastases and local recurrence has also been reported for primary benign retroperitoneal schwannomas [8,17].

## 4 Conclusion

Retroperitoneal schwannomas (RS) are a rare entity and the preoperative diagnostic workup represents a challenge, because image-morphological properties of these lesions are frequently unspecific and radiologic and nuclear medicine diagnostic procedures might be discrepant.

Deeper understanding of retroperitoneal schwannomas gained from preoperative radiologic diagnostics may serve to underline the need for targeted biopsy. This and its histopathological examination necessitate expert interventional radiologists and pathologists. Thus, even the pre-therapeutic diagnostic management should be performed in a specialized center. Correct diagnosis exerts an influence on the pre- and intraoperative therapy algorithm, thereby obviating the need for possible extended multivisceral resections.

If doubts remain regarding malignancy of the retroperitoneal lesion and findings are still controversial after thorough assessment, surgical oncologic resection represents the therapy of choice, if the surgical risk is adequate.

## Conflicts of interest

The authors declare no financial competing interests related to this work.

## Funding

There are no sponsors and there was no special funding for this case report. We acknowledge support from the German Research Foundation (DFG) and Leipzig University within the program of Open Access Publishing.

## Ethical approval

The ethical approval for the publication of this case was exempted by our institution because all of the data were collected from clinical records and imaging systems for routine perioperative planning.

## Consent

Written and informed consent was taken from the patient for publication of this case report and the accompanying images.

## Author contribution

Writing, editing, review of the literature: IG, SN, RT, CW.

Performed surgery: IG, SN, HS.

Performed diagnosis: SU, KTH, OS, KS, CW.

Editing: IG, RT, CW.

All authors have read and approved the manuscript.

## Registration of research studies

Not applicable.

## Guarantor

Prof. Dr. Ines Gockel, MBA.

## Provenance and peer review

Not commissioned, externally peer-reviewed.
